# Supplementary material for: Assigning metabolic rate measurements to torpor and euthermy in heterothermic endotherms: ‘torpor’, a new package for R
Source: Biol Open. 2022 Apr 4;11(4):bio059064. doi: 10.1242/bio.059064 (PMC9002798; doi:10.1242/bio.059064)
Supplement: Supplementary information [file biolopen-11-059064-s1.pdf]

Table S1. Model parameters and corroboration index for 28 datasets on small or medium-sized mammals described as either heterotherms or homeotherms.

Species	Type	*m*	*Autors* M_{TNZ}	*M* units	Authors T_{lc}	Authors T_t	Authors	*N*	T_{lc}	M_{TNZ}	*TMR*	T_t	β_t	α_e	β_c	α_c	α_r	*MR inhibit*	T_{be}	T_{bt}	PPO *TMR*	PPO M_r	Valid assignments	Corroboration	*M* assigned to torpor?
Unit	-	g	*M* units	-	°C	°C	-	-	°C	*M* units	*M* units	°C	*M* units/ °C	*M* units	**log(M unit)/ °C**	*M* units	*M* units	**%**	°C	°C	%	%	**-**	**-**	**-**
Callithrix pygmaea	Homeotherm	153	98	$mlO_2\ h^{-1}$	27.5 [8]	–	Genoud et al. 1997	27	29.15	102.97	NI	NI	-9.78	388.45	NI	NI	NI	NI	39.77	NI	89.80	86.80	0.63	1.00	No
Marmosa robinsoni	Heterotherm	122	0.8	$mlO_2\ g^{-1}\ h^{-1}$	26.5	–	McNab 1978	38	28.73	0.77	NI	NI	-0.10	3.49	NI	NI	NI	NI	36.41	NI	77.70	92.10	0.68	1.00	No
Phenacomys intermedius	Homeotherm	21.5	3.13	$mlO_2\ g^{-1}\ h^{-1}$	25.1 [6]	–	McNab 1992	75	25.48	3.09	NI	NI	-0.25	9.36	NI	NI	NI	NI	37.78	NI	80.00	94.60	0.85	1.00	No
Rattus niobe	Homeotherm	42.3	53.6	$mlO_2\ h^{-1}$	26	–	Genoud 2014	62	27.56	56.18	NI	NI	-4.56	181.97	NI	NI	NI	NI	39.97	NI	84.90	89.00	0.76	0.98	No
Sorex coronatus	Homeotherm	9.1	5.7	$mlO_2\ g^{-1}\ h^{-1}$	27.5	–	Sparti & Genoud 1989	33	22.61	5.94	NI	NI	-0.24	11.37	NI	NI	NI	NI	47.56	NI	95.20	97.20	0.97	0.81	No
Sorex minutus	Homeotherm	3.3	8.6	$mlO_2\ g^{-1}\ h^{-1}$	27.5	–	Sparti & Genoud 1989	28	25.36	8.67	NI	NI	-0.40	18.98	NI	NI	NI	NI	46.99	NI	95.60	95.00	0.89	1.00	No
Tolypeutes matacus	Homeotherm	1160	0.181	$mlO_2\ g^{-1}\ h^{-1}$	28	–	McNab 1980b	66	29.57	0.19	NI	NI	-0.04	1.24	NI	NI	NI	NI	33.94	NI	76.80	95.30	0.85	1.00	No
Cercartetus lepidus	Heterotherm	12.6	1.49	$mlO_2\ g^{-1}\ h^{-1}$	28.8	5.3	Geiser 1987	57	29.01	1.54	0.11	4.16	-0.20	7.47	0.08	0.08	0.95	0.49	36.72	4.68	4.60	33.50	0.98	1.00	Yes
Cercartetus nanus	Heterotherm	36	0.66	$mlO_2\ g^{-1}\ h^{-1}$	28.7	4.8	Song et al. 1997	171	26.15	0.64	0.06	2.86	-0.14	4.29	0.05	0.05	0.46	0.27	30.55	3.26	8.10	27.10	0.95	0.85	Yes
Cynopterus brachyotis	Heterotherm	37.4	1.27	$mlO_2\ g^{-1}\ h^{-1}$	29.7 [6]	–	McNab 1989	51	29.08	1.25	0.74	25.23	-0.21	7.18	NI	NI	NI	NI	34.38	28.76	44.80	94.30	0.76	0.92	Yes
Dasyuroides byrnei [1]	Heterotherm	118.2	0.7	$mlO_2\ g^{-1}\ h^{-1}$	28.3 [7]	15	Geiser & Baudinette 1987	75	26.13	0.64	0.38	12.97	-0.12	3.66	0.01	0.34	1.89	0.67	31.51	16.24	21.50	29.90	0.89	0.87	Yes
Eptesicus fuscus	Heterotherm	15	16.98	$mlO_2\ h^{-1}$	26.7	0	Willis et al. 2005	29	27.46	6.69	0.61	NI	-2.81	83.39	NI	NI	NI	NI	29.66	NI	13.60	80.50	0.93	1.00	Yes
Lasiurus cinereus [2]	Heterotherm	23.5 [4]	9	$mW\ g^{-1}$	30	–	Cryan & Wolf 2003	48	26.87	8.95	1.67	8.68	-1.09	38.23	NI	NI	NI	NI	35.15	10.22	24.70	78.00	0.90	0.81	Yes
Lasiurus seminolus	Heterotherm	9.4 [5]	1.26 [5]	$mlO_2\ g^{-1}\ h^{-1}$	32	5	Genoud 1993	204	30.69	1.43	0.12	6.27	-0.42	14.22	0.08	0.08	2.74	0.60	34.03	6.57	7.60	23.20	0.95	0.99	Yes
Macroglossus minimus	Heterotherm	16.3	1.29	$mlO_2\ g^{-1}\ h^{-1}$	30.9	20.4	Bartels et al. 1998	46	30.54	0.90	0.47	20.38	-0.16	5.89	0.06	0.16	3.78	0.93	36.32	23.35	27.50	26.30	0.65	0.97	Yes
Macroglossus minimus [3]	Heterotherm	16.4	0.88	$mlO_2\ g^{-1}\ h^{-1}$	31	21	Bonaccorso & McNab 1997	47	30.05	1.23	0.78	18.79	-0.22	7.75	NI	NI	NI	NI	35.78	22.39	30.50	79.50	0.91	1.00	Yes
Melonycteris melanops	Heterotherm	53.3	0.81	$mlO_2\ g^{-1}\ h^{-1}$	28	–	Bonaccorso & McNab 1997	30	28.84	0.81	0.44	17.05	-0.13	4.47	NI	NI	NI	NI	35.00	20.53	64.60	87.30	0.83	1.00	Yes
Natalus tumidirostris	Heterotherm	5.4	1.54	$mlO_2\ g^{-1}\ h^{-1}$	28	20	Genoud et al. 1990	30	27.22	1.46	0.82	19.00	-0.58	17.07	NI	NI	NI	NI	29.54	20.42	58.70	76.70	0.83	1.00	Yes
Nyctophilus geoffroyi	Heterotherm	8	1.42	$mlO_2\ g^{-1}\ h^{-1}$	34	–	Hosken & Withers 1999	84	32.32	1.43	0.24	8.62	-0.27	10.12	0.05	0.18	2.56	0.52	37.60	9.53	23.70	65.60	1.00	1.00	Yes
Peropteryx macrotis	Heterotherm	5.1	2.31	$mlO_2\ g^{-1}\ h^{-1}$	30.5	20	Genoud et al. 1990	49	35.01	2.50	1.80	20.36	-0.22	10.14	0.02	1.35	6.20	0.90	46.99	28.70	22.30	60.40	0.71	0.66	Yes
Pipistrellus pipistrellus	Heterotherm	4.9	7.6	$mlO_2\ h^{-1}$	33.2	0	Genoud & Christe 2011	95	32.79	7.62	0.54	2.22	-3.01	106.17	0.08	0.46	7.20	0.80	35.32	2.40	6.40	19.60	1.00	1.00	Yes
Planigale gilesi	Heterotherm	8.3	1.43	$mlO_2\ g^{-1}\ h^{-1}$	28.5 [8]	14	Geiser & Baudinette 1988	62	27.76	1.53	0.30	13.41	-0.37	11.65	0.07	0.13	5.19	0.55	31.93	14.22	19.90	34.40	0.89	1.00	Yes
Setifer setosus	Heterotherm	530	0.231	$mlO_2\ g^{-1}\ h^{-1}$	28	–	McNab 1980b	56	29.37	0.23	0.14	NI	-0.03	1.18	0.01	0.14	NI	0.88	36.20	NI	34.70	56.60	0.77	0.95	Yes
Sminthopsis macroura	Heterotherm	24.8	0.89	$mlO_2\ g^{-1}\ h^{-1}$	31.3	14	Song et al. 1995	155	30.80	0.84	0.18	16.39	-0.19	6.63	0.09	0.05	3.27	0.77	35.24	17.36	16.50	30.60	0.97	1.00	Yes
Steatomys pratensis	Heterotherm	27.5	1.315	$mlO_2\ g^{-1}\ h^{-1}$	28	20	Ellison 1995	39	26.73	1.24	0.26	16.38	-0.18	6.02	0.07	0.13	3.18	0.42	33.88	17.85	21.60	45.40	0.77	1.00	Yes
Syconycteris australis	Heterotherm	17.8	1.44	$mlO_2\ g^{-1}\ h^{-1}$	29.5	18	Geiser et al. 1996	91	32.13	1.27	0.49	15.11	-0.14	5.78	0.05	0.24	2.61	0.90	41.31	18.62	21.60	28.60	0.88	0.95	Yes
Syconycteris australis [1]	Heterotherm	18	1.38	$mlO_2\ g^{-1}\ h^{-1}$	30.2	18	Coburn & Geiser 1998	93	27.53	1.49	0.61	16.37	-0.17	6.00	0.07	0.23	3.33	0.86	36.22	20.07	32.00	43.10	0.85	0.97	Yes
Zaedyus pichiy	Heterotherm	1740	0.226	$mlO_2\ g^{-1}\ h^{-1}$	28	–	McNab 1980b	57	31.82	0.23	0.15	22.49	-0.02	0.94	0.02	0.11	0.65	0.82	42.25	29.47	29.30	71.70	0.70	0.75	Yes

Subset of published data considered: [1]=summer group; [2]=males; [3]=lowland population.
m=average body mass; Autor's M_{TNZ}=author's estimation of metabolic rate within *TNZ*; *M* units=units for metabolic rate; Authors' T_{lc} and T_t=author's estimation of lower critical temperature and threshold ambient temperature for torpor, respectively. When *m*, M_{TNZ} or T_{lc} were not explicitly provided, they were: [4]=back-calculated from M_{TNZ} in % of expected from body mass; [5]=calculated as a weighted average of two seasonal groups; [6]=extracted from a graph; [7]=calculated as the intercept of the euthermic line and M_{TNZ} given by the authors; [8]=obtained as the mean of a given range; *N*=number of digitized data points (sample size); Model parameters are defined in text.
NI=Parameter considered as not identifiable.

Biology Open • Supplementary information
